# The Effects of Thermal Treatment on Lipid Oxidation, Protein Changes, and Storage Stabilization of Rice Bran

**DOI:** 10.3390/foods11244001

**Published:** 2022-12-10

**Authors:** Muhammad Tayyab Rashid, Kunlun Liu, Simeng Han, Mushtaq Ahmed Jatoi

**Affiliations:** 1 College of Food Science and Engineering, Henan University of Technology, Zhengzhou 450001, China; 2 School of Food and Strategic Reserves, Henan University of Technology, Zhengzhou 450001, China; 3 Department of Botany, Shah Abdul Latif University, Khairpur 66020, Pakistan

**Keywords:** rice bran, dry heat treatment, amino acid profile, SDS-PAGE, malondialdehyde

## Abstract

Rice bran is a nutrient-rich and resource-dense byproduct of rice milling. The primary cause of rice bran utilization limitation is oxidative deterioration and inadequate storage facilities. Improving stability to extend the shelf-life of rice bran has thus become an utmost necessity. This study aimed to stabilize raw fresh rice bran (RB) by using dry heat methods at 120 °C (233, 143, and 88 min) and 130 °C (86, 66, and 50 min). The results indicated that after dry heat pretreatment, peroxidase levels were at 90%, and the storage stability of dry-heat-stabilized RB was better. However, with an increase in treatment temperature and time, the peroxidase activity improved while the lipase activity decreased to a certain extent without significant changes. The total saturated and unsaturated fatty acids were significantly unchanged during storage, while oleic/linoleic acid increased substantially by 1% at 120 °C for 88 min. The increase in treatment time and temperature was beneficial in controlling the fatty acid values. However, extended treatment time caused an increase in the peroxide value and MDA. The essential and non-essential amino acid ratios, which evaluate a protein’s nutritional value, remained relatively stable. The essential subunit of rice bran protein was not affected by the temperature and time of dry heat treatment and storage time.

## 1. Introduction

Rice bran (RB) (rice milling byproduct) mainly comprises protein (11.3–14.9%), carbs (mostly starch, 34.0–62.0%), moisture (7.24–10.63%), ash (12.26–14.01%), and oil (15.0–19.7%) [1]. These components are in compact forms in rice cells, such as liposomes, amylases, and proteosomes [1]. RB’s traditional uses include animal feed, oil extraction, and boiler fuel [2]. In addition to being a source of essential nutrients, such as protein, carbohydrates, oil, dietary fiber, and phenolic compounds, it can be used as an active ingredient in foodstuffs [3]. Due to RB’s high fiber and oxidative rancidity, it is rarely ingested as a whole food [4]. RB protein is more abundant and hypoallergenic than casein and soy proteins [5]. As a result, RB protein is projected to have a high potential for use as a functional component in food applications, particularly in infant food formulations [6].

However, RB protein is scarce due to extraction issues and low purity. Disulfide linkages in RB proteins make them insoluble [7]. Heat stabilization of RB is a strategy for preventing the breakdown of oil and inactive lipoxygenase, which typically results in protein denaturation and the strengthening of the bonds between carbohydrates and proteins or other components [2]. RB is susceptible to specific physicochemical changes quickly after separating from the grain due to the breakdown of lipids by enzymes such as lipase. As a result, the stabilization approach necessitates slowing or stopping harmful alterations [8]. There have been numerous approaches to RB stabilization developed, including toasting [9], extrusion [10], autoclaving [11], microwave treatment [12], ohmic heating [13,14], hydrothermal treatment [15,16], infrared radiation [17,18], super-heated steam heating [19], chemical stabilization or refrigeration [20], acid treatment [21], radio-frequency [22], infrared-vacuum treatment [23], and dry heat [24]. Dry heat treatment can inactivate lipase for stabilization, primarily by high-temperature treatment. The dry heat stabilization method is simple, sound, and economically feasible; hence it has been widely reported [10]. The associated benefit of dry heat stabilization is that it has no adverse effects on protein quality (Sharma et al., 2004). However, there are growing concerns regarding the impact of dry heat treatment on the functional characteristics of RB protein. Currently, there are limited studies on the dry heat steady-state effects along with the storage period of rice bran. This paper systematically studies the lipid oxidation and protein changes of rice bran during the storage period of dry heat treatments.

Even though RB is rich in protein, lipids, and antioxidants, it is neglected as a raw ingredient for nutritious meals and pharmaceutics. The primary causes of this are oxidative and hydrolytic rancidity. Glycerol molecules that are hydrolytically rancid have lost their ability to hold onto fatty acids, resulting in oxidative rancidity. Enzymes produced by the RB are crucial to properly controlling the process. Lipase catalyzes the hydrolysis reaction, occurring swiftly after rice milling, and yields glycerol and free fatty acids (FFA) from triglycerides. The subsequent FFAs are harmful compounds making RB inappropriate for human ingestion [25]. Thus, RB stability is essential since fat is rapidly oxidized, and lipid peroxides can negatively affect animals and humans.

In this context, in the present study, rice bran byproduct obtained from brown rice milling was stored for 60 days and stabilized with two dry heat treatments, i.e., 120 (233, 143, and 88 min) and 130 °C (86, 66, and 50 min). The effects of lipid oxidation, residual peroxidase activity, lipase activity, free fatty acid, fatty acid composition, amino acid composition, and protein alterations were examined during the storage period of RB. This article could provide basic information about the physicochemical properties of such isolates, which could aid in determining their application in foods.

## 2. Materials and Methods

RB (source: Yuanyang Basu Rice Industry Co., Ltd., China) was obtained by dehulling and polishing paddy rice under laboratory conditions. Rice bran was stabilized by dry heat treatment immediately after purchase, as described below. Disodium hydrogen phosphate, citric acid, sodium bisulfite, potassium hydroxide, and ethanol were obtained from Luoyang Chemical Reagent Factory (Luoyang, China). Hydrogen peroxide, potassium thiocyanate, N-Hexane, trichloromethane, trichloroacetic acid, and ferrous chloride were bought from Tianjin Kaidi Chemical Reagent Co., Ltd. (Tianjin, China). The other chemical reagents were pure and of analytical grade.

### 2.1. Dry Heat Treatment of RB

RB was evenly distributed in thin layers (1 cm) in shallow open pans [3,6] and preheated in a drying oven set at 120 °C (for 233, 143, and 88 min) and 130 °C (for 86, 66, and 50 min). The temperatures and exposure time levels were selected based on preliminary tests. Pretreated RB was placed in a desiccator to attain a relative humidity of 70%. The RB was packed in polyethene bags and then stored in a biochemical incubator (HWS-250 Shanghai Jinghong Experimental Equipment Co., Lt, Shanghai, China) at 25 °C for 60 days. 

### 2.2. Determination of Peroxidase Residual Activity (PRA) in RB

The PRA activities were measured using the procedure outlined by Tan et al. [26]. The RB enzyme was extracted, and p-nitrophenol (p-NP) as a substrate solution was mixed and incubated at 37 °C for 5 min. The absorbance of all the samples was monitored at 470 nm after 1 and 3 min of reaction time, respectively.

### 2.3. RB Lipase Activity (LA)

The LA was determined using a spectrophotometric assay (722 s, Shanghai Yidian Analytical Instrument Co., Ltd., Shanghai, China) [27,28]. The absorbance was recorded at 405 nm.

### 2.4. Free Fatty Acid Analysis (FFA)

The FFA of RB was performed using the AOAC technique [29]. Specifically, 5 g of RB was agitated in 40 mL of ethanol/diethyl ether (20:20 *v/v*). After stirring for 10 min on an orbital shaker, the resultant mixture was shifted to a test tube followed by centrifugation (5 min, 6797× *g*, 25 °C). Then, the subsequent supernatant was titrated with 0.05 mol L^−1^ KOH.

### 2.5. Peroxide Value (PV)

PV was analyzed following the AOCS Official Method Cd 8–53 [30] and represented as meq.kg^−1^.

### 2.6. Malondialdehyde (MDA) Analysis

MDA was detected by Parrado et al. [31]. The samples were heated to 100 °C for one hour, followed by TBA reagent treatment (20 mM TBA in 50% *v/v* glacial acetic acid). After cooling, the mixture was mixed with butanol, and the subsequent organic layer was drained. The fluorescence was monitored at λex = 515 nm and λem = 585 nm.

### 2.7. Fatty Acid Composition (FAC) Analysis

The FAC of RB was estimated by an Agilent 1100 gas chromatography system (Agilent Technologies, Santa Clara, CA, USA), along with a DB-23 (50% cyanopropyl-methylpolysiloxane) capillary column (60 m, 0.25 mm i.d., 0.25 m film thickness, Agilent, Waldbronn, Germany). A standard fatty acid methyl ester (FAME) was formulated with 0.5 mL methanolic potassium hydroxide. The results were presented in relative area % [17].

### 2.8. Crude Protein Content Analysis

The crude protein content (*n* × 5.95) was measured using the Kjeldahl digestion technique [32].

### 2.9. Amino Acid Composition (AAC) Analysis

RB samples were hydrolyzed using 6 M HCl and 3% phenol (*w/v*) in vacuum glass tubes (22 hr) and then exposed to an amino acid analyzer (S-433D, Shimadzu, Kyoto, Japan) equipped with an ion-exchange column (Shim-pack Amino-Na, 6 100 mm). After column separation, the resultant amino acids were tagged with o-phthalaldehyde and identified using a fluorescence detector (Ex: 350 nm; Em: 450 nm). The amino acids were identified by comparing them with the amino acid profile.

### 2.10. SDS-PAGE (Sodium Dodecyl Sulfate-Polyacrylamide Gel Electrophoresis)

SDS-PAGE of the samples was examined, as reported by Li et al. [33]. The acrylamide gels contained a 10% separating gel, 5% stacking gel, and 0.1% SDS. The 1% samples with varying electrocatalytic durations were combined in a buffer (0.0625 M Tris–HCl, 10% glycerin, 10% SDS, 5% 2-mercaptoethanol, and 0.0025% bromophenol blue), and heated in a boiling water bath for 5 min. After cooling, the samples were placed into the gels. Coomassie Brilliant Blue G-250 was used to dye the gel. After staining, the gel was decolorized for 4 h. Electrophoresis was used to capture the gels (DYCA-25 D, Beijing Liuyi Biotechnology Co., Ltd., Beijing, China).

### 2.11. Statistical Analysis

The experiments were performed in triplicate, representing the findings as the mean ± SD. SPSS (16.0) was used to analyze the test data statistically. A one-way ANOVA was performed to assess the analysis. Duncan’s multiple range test was used for comparison at the 95% confidential level (*p* < 0.05).

## 3. Results and Discussion

### 3.1. Changes in PRA of Dry-Heat-Treated RB during Storage

**Figure 1:**
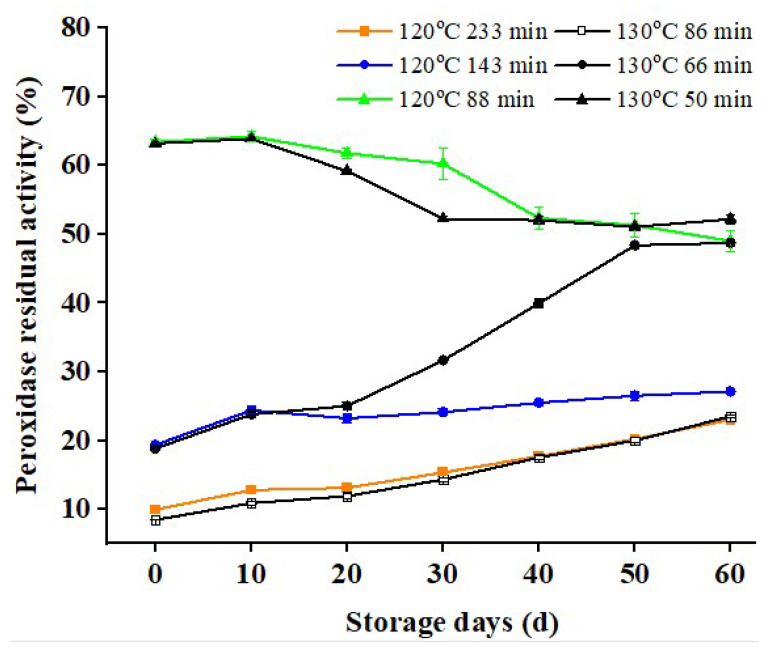
Changes in the residual peroxidase activity of rice bran after dry-heated treatment.

**Figure 2:**
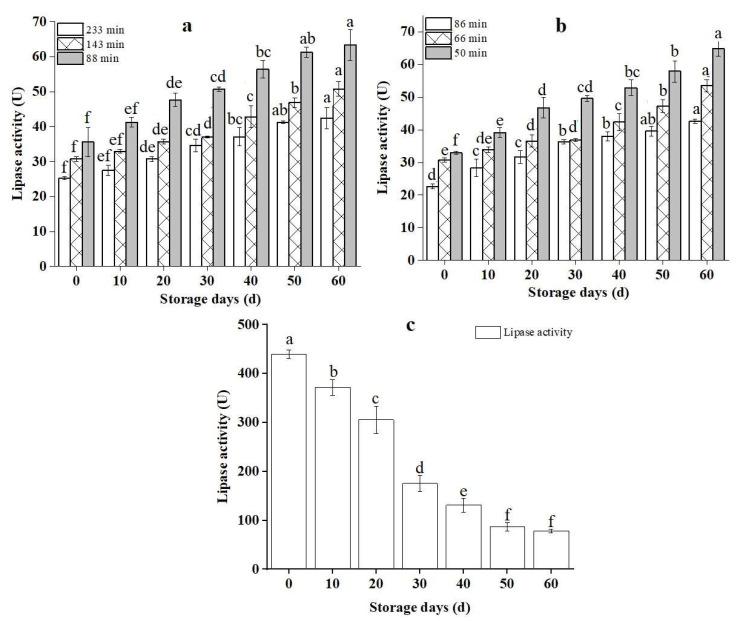
Changes in lipase activity of rice bran after dry heat treatment; (**a**,**b**) refer to the treatment temperature of rice bran at 120 °C and 130 °C; (**c**) refers to untreated rice bran. **Note:** The lowercase letters refer to the significant difference of the same sample for different storage periods at the same temperature and treatment conditions at *p* < 0.05. CNT: Control.

Figure 1 depicts the variations in PRA of RB treated with different dry heat conditions within 60 days of storage. It can be seen from Figure 2 that the PRA in RB can be the same after being treated at 120 °C and 130 °C for a certain period, but it has different trends during storage.

Peroxidase residual activity decreased slowly at 120 °C for 88 min and 130 °C for 50 min, whereas it increased at 120 °C for 143 min and 233 min and 130°C for 66 min and 86 min, indicating an increasing trend in PRA. Gong et al. [25] also observed a slight rise in RPA at 110 °C during the stability of fresh RB. The PRA in RB treated at 120 °C for 233 min, and 130 °C for 66 min increased considerably after 30 days of storage and peaked at 60 days. The increase in peroxide samples indicated that heat pretreatment was effective in delaying enzyme degradation. These findings imply that heat pretreatment has a practical impact on enzyme inactivation. Yan et al. [28] discovered that the PRA of IR-treated RB enhanced slightly on the 10th day during the 20-day storage duration.

Comparing the changes in PRA in RB treated at different temperatures during the storage period, even when the treatment temperature was similar, the treatment time significantly influenced RB’s stability over time. After 60 days of storage, residual enzyme activity in radiofrequency-treated RB and the control samples was enhanced [34,35]. The storage stability of RB differed significantly when the same treatment time was applied at different temperatures (the higher the temperature, the stronger the storage stability). This is because the peroxidase in RB has a strong heat resistance and is not easy to be inactivated. However, the inhibitory effect became uncertain with the prolonged storage time and the reabsorption of moisture by RB. The residual enzyme activity of RB with low peroxidase residual activity heightened gradually in the primary storage stage [25]. RB with high PRA was not affected by moisture during storage and maintained its high PRA level. Due to the limitation of the fat content of RB, the PRA of RB decreases slowly with the prolongation of storage time [36].

### 3.2. Dry-Heat-Treated RB’s Lipase Activity Changes

RB has a short shelf-life due to the conversion of lipase hydrolyzes triglycerides into FFAs [37]. Therefore, the LA can be used to assess the storage stability of RB. Within 60 days of storage, the variations in the LA of RB samples treated with various dry heat treatments are shown in Figure 2. The LA was significantly reduced after dry heat treatment, with a reduction of at least 13 times that of the control sample (CNT). As demonstrated in Figure 2a, the LA of dry-heat-treated rice bran at 233 min was lower than at 143 and 88 min. Furthermore, depending on the storage duration, the LA of rice bran reduced steadily over time. Boonmawat et al. [19] found a significant decrease in the LA in RB control samples. The elevated temperature associated with the dry heating conditions probably reduced LA. The LA of the treated RB increased as the storage period progressed but was significantly lower than control samples after 60 days, as shown in Figure 2.

This is because some inactivated lipases gradually recovered their vitality but played a limited role during storage, indicating that dry heat treatment effectively diminished the activity of RB lipase by maintaining the stability of dry heat RB during storage [38]. It also revealed that lipase was damaged after 15 min of heating at 100 °C, while lipoxygenase was inactivated after 10 min of heating at 50–70 °C [37]. When the variations in RB LA were compared, it was evident that even at the same treatment temperature, the treatment duration considerably impacted the RB stability during storage. The LA fluctuated significantly across all samples, with significant changes observed at each storage time. In Figure 2b, the lipase activity in rice bran did not deviate significantly in the treatment duration, regardless of the storage period. The treatment timing follows the same pattern as in Figure 2a. Even though a significant decrease was observed in the storage period from 0 to 60 days, the reduction speed of 50 min treatment was significantly (*p* < 0.05) faster than 66 and 86 min in the storage period. In contrast, the LA of untreated rice bran in Figure 2c decreased considerably from 0 to 50 days and then was constant to 60 days of storage. It was higher than the dry-heat-treated samples after 60 days of storage. The LA dropped dramatically as the treatment time increased. Li et al. [39] achieved similar results by applying infrared (IR) radiation on wheat germ and observed that the LA of wheat germ reduced (*p* < 0.05) with administering temperature and time. Different temperatures and the same treatment time have the same effects on lowering RB lipase activity, indicating that lipase is temperature sensitive and its activity can be significantly reduced at a lower temperature [39]. RB lipase has weak heat resistance and is quickly inactivated. Heat treatment can be represented as an efficient method of rendering lipase inactive in raw and brown rice while reducing the rice kernel’s moisture [40,41]. Lipase is more sensitive to processing temperature and time than peroxidase activity. Similarly, the lipase activity of control samples gradually decreased during the storage period due to the influence of the external environment, e.g., light, temperature, and oxygen [42]. However, the LA was significantly higher than dry-heat-treated RB during storage.

### 3.3. FFA Changes in Dry-Heat-Treated RB

**Figure 3:**
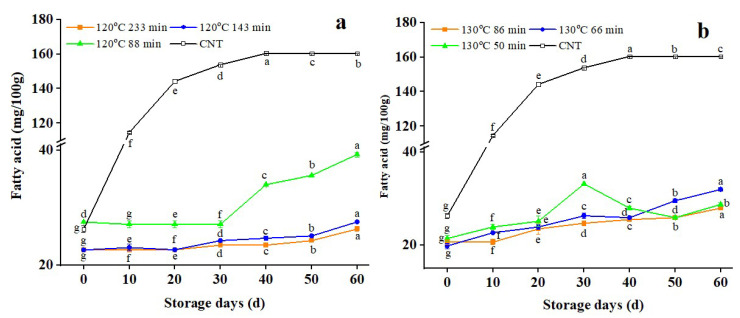
Changes in fatty acid values of rice bran after dry-heated treatment. **Note:** The lowercase letters refer to the significant difference of the same sample for different storage periods at the same temperature and treatment conditions at *p* < 0.05. CNT: Control.

Figure 3 demonstrates the variations in FFA values of RB treated at 120 °C and 130 °C for different time intervals and the control samples at 25 °C for 60 days.

The FFA value of control samples increased during storage (*p* < 0.05) and then tended to flatten out after reaching a peak after 40 days, confirming that the stabilizing treatment successfully prevented enzymatic degradation. A similar increasing trend of FFAs was noted by Lv et al. [6] for rice bran during the storage period. The FFA values before and after storage were 26.283 and 160.287 mg/100 g, respectively. The FFA value of RB increased rapidly during the first 10 days, then slowed down, but the overall trend continued to rise, consistent with the law of rancidity and the deterioration of fresh RB. According to Sharma et al. [10], the increasing rate of FFAs is significantly impacted by the stabilizing procedures and storage time. Lipase is activated by rice milling and fat contact and can rapidly hydrolyze RB fat at 25 °C, causing the FFA value to rise quickly within a few hours. In Figure 3a, the fatty acid value of RB after dry heat treatment at 120 °C for 233 min, 143 min, and 88 min increased by 16%, 21%, and 43%, respectively, after storage for 60 d. The FFA value of RB after dry heat treatment at 130 °C for 86 min, 66 min, and 50 min increased (*p* < 0.05) by 35%, 61%, and 34%, respectively, during 60 days of storage in Figure 3b, this expansion in FFA content might result from the lipase enzyme’s residual lipolytic activity that was raised under satisfactory storage conditions.

Similarly, Lavanya et al. [37] found that under comparable conditions, the FFAs in untreated RB could be retained in Ziploc bags for 6 weeks of storage. According to Lakkakula et al. [14], they preheated the RB in a microwave oven (3 min) before loading and increased FFA after 6 weeks of storage at ambient temperature. After storage for 60 days, the FFA value of the control sample was about six times higher than before storage, while the dry heat treatment effectively reduced the FFA content of RB. The increment in FFA of RB (treated at 120 °C for different time intervals) decreased dramatically (*p* < 0.05) as the treatment time was increased. RB treated at 130 °C for 86 min caused an increase in FFA value of around 81% compared to RB treated at 120 °C for 88 min. This demonstrates that elongating the heat treatment of the RB leads to a better lipase inactivation effect and prohibits the growth of FFAs. Both the LA and peroxidase activity influenced the degree of rancidity of RB. Therefore, the dry heat treatment temperature and time should not be too low to measure the stabilization effect of RB [43,44]. Otherwise, RB’s nutritional components and functional properties will be affected, which is not conducive to the full utilization of RB.

### 3.4. Changes in Peroxide Value of Dry-Heat-Treated RB

**Figure 4:**
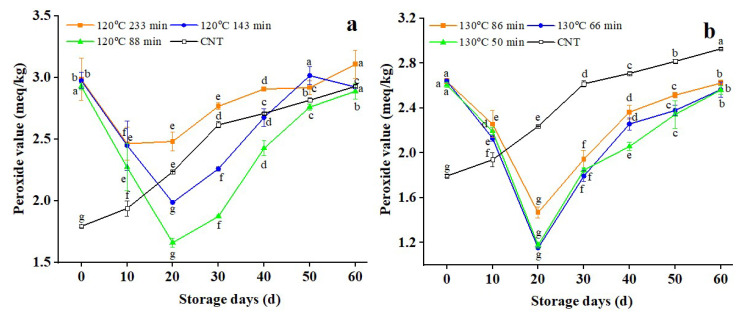
Changes in the peroxide values of rice bran after dry-heated treatment. **Note:** The lowercase letters refer to the significant difference of the same sample for different storage periods at the same temperature and treatment conditions at *p* < 0.05. CNT: Control.

The unsaturated fatty acids generated by hydrolysis are oxidized to peroxide under lipoxidase. It can be further oxidized and decomposed into aldehydes, ketones, and other substances. However, there are limited studies on the dry heat treatment effects on the oxidative rancidity of RB during storage. The heating procedure decreases the fat content of RB, reducing the level of FFA and the peroxide value [45]. The lowest FFA and PV levels are the best quality parameters since they reflect minimal fat degradation during storage [35]. Figure 4 shows that the peroxide value of untreated RB increases during storage, while RB treated at 120 °C and 130 °C for various times decreases at first, then increases.

The heating process reduces the fat content of the rice bran, thus reducing the peroxide value [45], and when the free fatty acid content is high, the peroxide value rises as well [35]. The dry heat treatments efficiently reduce (*p* < 0.05) the peroxide content to some extent; hence the peroxide value of RB in the control samples after 60 days was roughly 1.6 times that before storage. However, the initial peroxide value of RB after different dry heat treatments was significantly (*p* < 0.05) more significant than the untreated RB, which was caused by heat treatment promoting fatty acid oxidation and peroxide production. These findings are consistent with earlier research that used super-heated steam at 170 °C, infrared at 700 Watts, dry-heating, and hot-air-assisted radio frequency at 90 °C to lower the PV of RB [35,45,46], respectively. The peroxide value of RB increased significantly (*p* < 0.05) after 20 days of storage, which was caused by the further oxidation and decomposition of peroxide into aldehydes and ketones.

Peroxide formation was more significant (*p* < 0.05) as storage time increased, with an increasing trend compared to its decomposition rate. In Figure 4a, the PV of RB treated at 120 °C for 143 min and 88 min increased faster than that of RB treated at 233 min after 20 days of storage. However, after 20 days of dry heat at 130 °C in Figure 4b, the peroxide value of RB (with different treatment times) was gradually lower than that of CNT, demonstrating that a higher temperature and the extended duration of the dry heat treatment slightly altered the peroxide values. These findings indicate that heat pretreatment may also influence enzyme inactivation. According to the CODEX guidelines, the determined degree of PV in edible oils and fats is 15 meq/kg [47]. The peroxide values reported in the current study were lower than the CODEX standards. Generally, the samples exhibited lower peroxide values and a small degree of lipid oxidation in the early storage stage. With the increase in storage time, the peroxide generation rate was more significant (*p* < 0.05) than its degradation rate, so the peroxide value increased. The slower and consistent growth in peroxide value indicated that the lipase and peroxidase enzymes had been inactivated, and no free radicals were available for further degradation [48]. Dry heat treatment accelerated the oxidative rancidity of RB, demonstrating the importance of temperature and treatment time in improving storage stability. As a result, if the temperature is too high and the length of dry heat treatment is too long, the peroxide value of RB will always be greater than that of untreated RB.

### 3.5. Changes in Malondialdehyde (MDA) Content

**Figure 5:**
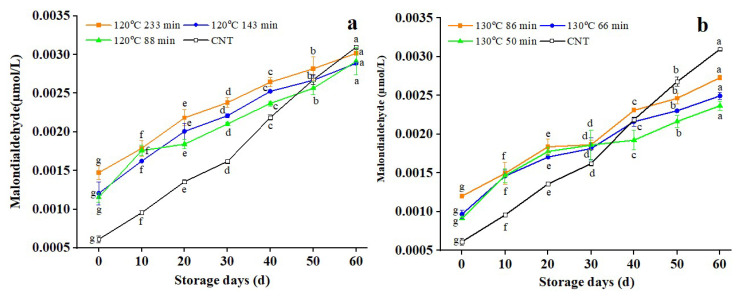
Changes in malondialdehyde content in rice bran after dry-heated treatment. **Note:** The lowercase letters refer to the significant difference of the same sample for different storage periods at the same temperature and treatment conditions at *p* < 0.05. CNT: Control.

Figure 5 shows that the malondialdehyde content of untreated RB increased throughout the storage period. The increase was gradual during the first 30 days of storage and then accelerated in the final 30 days. FFAs accumulate in large quantities during storage, causing fat oxidase enzyme activation that leads to fat oxidation, producing hydroperoxides [49]. The late storage period is the stage of MDA accumulation when the content of MDA increases rapidly [50]. Initially, the MDA of the dry-heat-treated RB was significantly affected by the untreated RB sample, caused by the heat treatment to promote the oxidation of FAs, which in turn produced peroxides, and thus further oxidized and decomposed into aldehydes and ketones. The MDA increased quickly with the increasing storage period (0–30 days) because the peroxide accumulated in the early stages deteriorated rapidly. After 30 d of storage, as peroxide’s decomposition rate decreased, the MDA content increased slowly. As demonstrated in Figure 5, the MDA contents of RB treated at 120 and 130 °C were substantially greater than the untreated samples. However, the peroxide value of RB with different treatment times at 130 °C was considerably lower than untreated samples after 50 days’ storage. Thus, the trend of RB peroxide was strongly tied to the malondialdehyde level, which is likewise an unavoidable byproduct of lipid oxidation.

### 3.6. Effect of Dry-Heat-Treated on Fatty Acid Composition (FAC) and Quantity of RB

The variations in fatty acid composition and dry-heat-treated RB content throughout storage are tabulated in Table 1. The FAC of RB consists mainly of eight types, most unsaturated fatty acids, including 80% oleic acid and linoleic acid [51,52]. Linoleic and oleic acids are essential for overall good health [4]. Different fatty acids respond differently to the temperature and time of the dry heat treatment. The changes in fatty acids also varied under other dry heat treatments. Some of the fatty acids of RB after dry heat treatment had no significant differences, and some had substantial differences but no clear trend, which proved that the oxidative rancidity of RB during dry heat treatment is a dynamically changing process. These findings might be attributed to the high temperatures used in heat treatments, which cause alterations in the fatty acid composition and characteristics of the materials [53].

With the escalating storage, the oleic acid (18:1) content increased, while the range of linolenic acid (18:2, 18:3) decreased significantly during the storage of untreated RB. Linoleic acid was the most copious unsaturated fatty acid (UFA) and was more prone to oxidative rancidity than monounsaturated fatty acids such as oleic acid [54]. Additionally, it has been asserted that reducing UFAs results in an unpleasant taste [55]. During the increased storage periods, the oleic acid content was increased with elevating temperatures for short-dry-heat-treated samples. In long dry heat treatments, no significant change was observed for oleic acid. Hence, the different durations of dry heat treatments influence the stability of RB samples. However, dry heat treatment did not significantly affect RB’s total saturated and unsaturated fatty acids. Likewise, Chotimarkorn et al. [38] revealed insignificant changes in FAC during storage. In addition, the oleic acid/linoleic acid ratio (O/L) is an indicator to measure the stability of fatty acids to oxidative rancidity. Therefore, a high ratio generally represents a good storage performance. The oleic acid/linoleic acid ratio of dry-heat-treated RB increased significantly during storage compared to untreated samples. Thus, the storage stability of heat-treated RB samples was relatively higher to a certain extent.

### 3.7. Changes in Crude Protein

RB has a considerable amount of protein. Dry heat treatment and long-term storage of RB have non-significant impacts on the protein content. Similarly, Sharma et al. [10] revealed that dry heat stabilization does not affect the quality of protein. The experimental crude protein content ranged from 12 to 20%, comparable with Watchararuji et al. [7], who investigated RB protein using high-performance liquid chromatography. Table 2 shows the changes in crude protein content of different dry-heat-treated RB during storage. No significant disparity was found between untreated and treated RB samples, demonstrating that dry heat treatment has no discernible influence on crude protein content. Furthermore, the crude protein content of RB did not correlate considerably with storage time [56].

### 3.8. Changes in the Amino Acid Composition (AAC)

Table 3 shows the RB’s AA composition changes at 120 and 130 °C of 0 and 60 days. In contrast, the comparison of the control group and detailed storage days are presented in Supplementary Data Tables S1–S3. The table comparison showed that the amino acid content of untreated and dry-heat-treated RB changed significantly during the storage period. Amino acids (AAs) showed both upward and downward trends. Therefore, no consistent trend was found between the different treatment groups of RB amino acids. The varying amounts of AAs might be attributed to the extraction method or alkaline treatment [57]. The contents of serine and proline in untreated RB decreased while valine and leucine increased significantly (*p* < 0.05) as the storage time increased. No significant variation was observed in the content of alanine, isoleucine, tyrosine, phenylalanine, histidine, lysine, and arginine. These AAs play an essential role in the metabolic processes of organisms and play a vital role in various chemical reactions, which enhance the function of human cells and organs and make it beneficial for the body’s health. The typical limiting AAs of grains are lysine and threonine [2]. Still, the content of cysteine and methionine in this study was lower than that of lysine because the AAs were determined using the acid hydrolysis method, which partially destroyed them. Generally, lysine is the main restrictive AA of many crops [58]. Hence, dry heat treatment affects the AA composition and content of RB. Eker et al. [59] and Pietrysiak et al. [60] found that the AA content decreased significantly when storing peanut and pea-rice. The AA content of RB treated at the same temperature reduced considerably with increased treatment time. However, the AA content of dry-heat-treated RB changed to differing degrees during storage. The prolonged dry heat treatment time led to a minor change in AA content, indicating that it affects and improves the AA storage stability to a certain extent. The content of total AAs, essential AAs, and non-essential AAs changed before and after dry heat treatment during the storage period in the current study. Although RB’s total AA content has decreased, the E/T and E/N that measure the nutritional value of protein were found to be relatively stable. A protein’s amino acid composition in RB is closer to the WHO/FAO standard model for essential AAs from a nutritional perspective [61]. High-quality RB protein can be employed as a dietary supplement because of its nutritional benefits [60]. In the current investigation, no consistent change in AA concentration was observed due to varied treatment and storage conditions.

### 3.9. Electrophoresis Analysis

**Figure 6:**
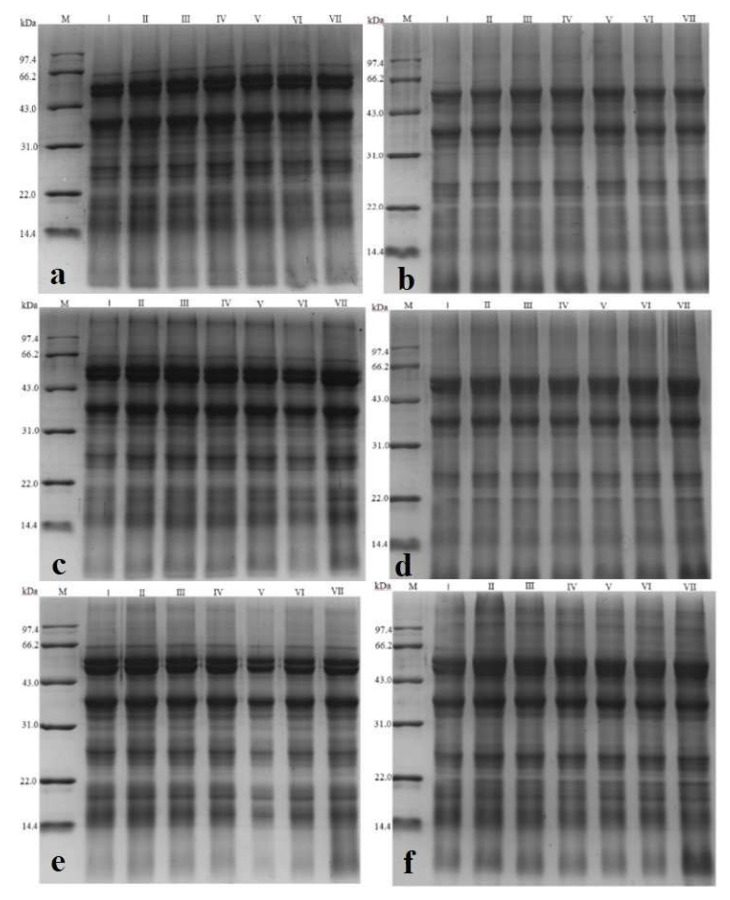

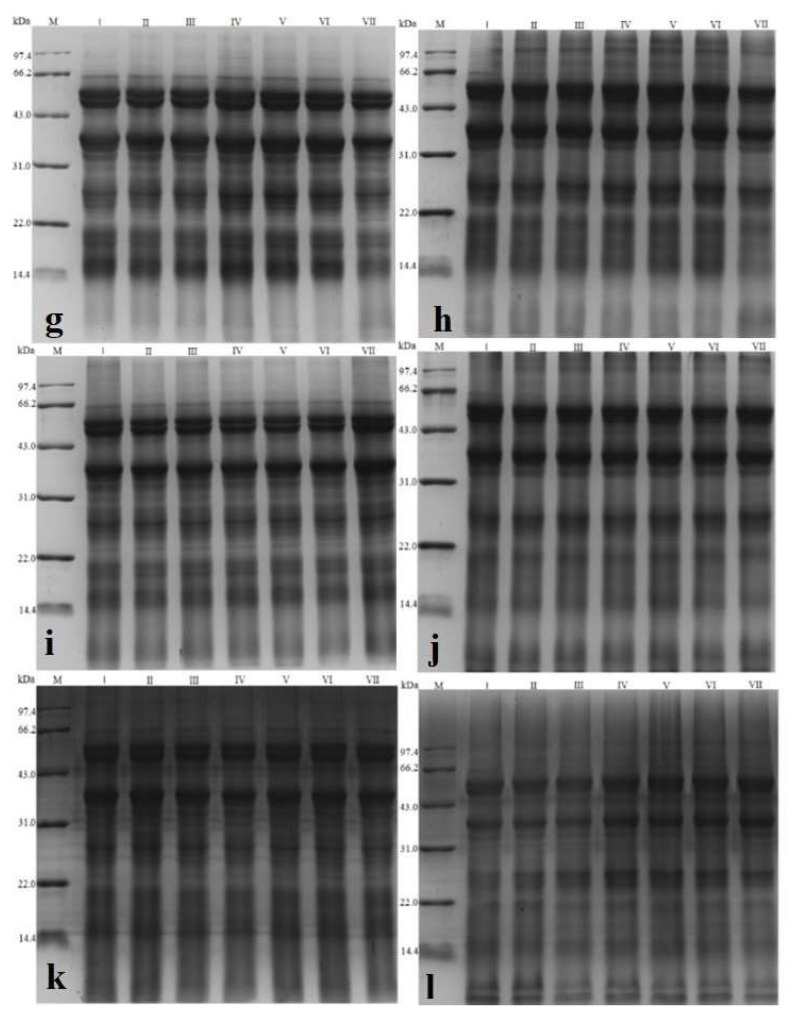

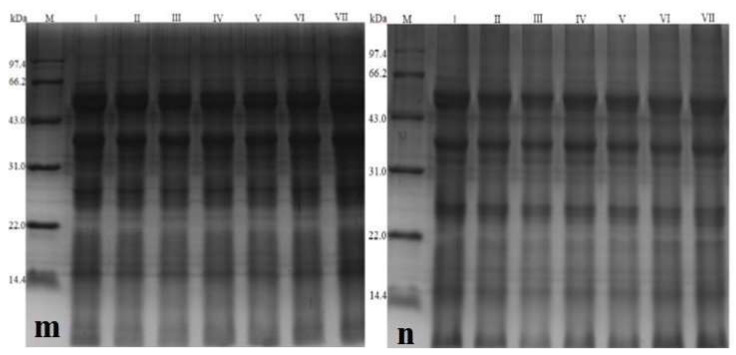
Sodium Dodecyl Sulfate-Polyacrylamide Gel Electrophoresis (SDS-PAGE) of dry-heat-treated rice bran protein. (**a**,**b**) represent non reduced electrophoresis and reduced electrophoresis analysis of untreated rice bran (RB) protein, respectively; (**c**,**d**) are expressed as non-reductive electrophoresis and reductive electrophoresis analysis of RB protein treated at 120 °C for 233 min, respectively; (**e,f**) are expressed as non-reductive electrophoresis and reductive electrophoresis analysis of RB protein treated at 120 °C for 143 min, respectively; (**g**,**h**) are expressed as non-reductive electrophoresis and reductive electrophoresis analysis of RB protein treated at 120 °C for 88 min, respectively; (**i**,**j**) are expressed as non-reductive electrophoresis and reductive electrophoresis analysis of RB protein treated at 130°C for 86 min, respectively; (**k**,**l**) are expressed as non-reductive electrophoresis and reductive electrophoresis analysis of RB protein treated at 130 °C for 66 min, respectively; (**m**,**n**) are defined as non-reductive electrophoresis and reductive electrophoresis analysis of RB protein treated at 130 °C for 50 min, respectively; Lanes I–VII represent RB samples stored at 25 °C for 0, 10, 20, 30, 40, 50, and 60 days, respectively.

SDS-PAGE is a popular technique for determining molecular protein weight and hence was adopted here to observe the changes in dry-heat-treated RB samples. Figure 6 shows the electrophoretic analysis of RB protein under different dry heat treatments; the non-reduced electrophoretic analysis is represented by a, c, e, g, i, k, and m, and the reduced electrophoretic analysis is represented by b, d, f, h, j, l and n. The difference between the two types of electrophoresis is that the disulfide bond of the RB protein is not opened in the non-reducing electrophoresis. The RB protein is destroyed in the reducing electrophoresis. Polyacrylamide gel electrophoresis (PAM) technology separates proteins according to their molecular weight under the action of an electric field. Some studies have analyzed that the molecular weight of RB protein is mainly distributed in the regions of 43.0–97.4 kDa, 20.1–43.1 kDa, and less than 20.1 kDa [1]. According to the solubility of RB protein in water, salt, alcohol, and alkali solutions, it can be divided into albumin, globulin, glutenin, and gliadin [2], and the proportion of these four proteins is approximately 37:36:22:5. The molecular weights of these four RB subunits varied from 30–45 kDa to 20–66 kDa, 10–66 kDa, and 10–53 kDa. These findings support Ling et al. [62], who discovered that the molecular weight of RB protein subunits ranged from 10 to 55 kDa. RB’s soluble protein content values were also consistent with soybean protein [63]. Protein molecules with small molecular weights move faster than those with large ones. In a vertical gel, the molecular weight of proteins decreases sequentially from top to bottom. According to the reduced electrophoresis in Figure 6, RB protein’s primary molecular weight distribution was about 64 KD, 38 kD, and 24 kD. These essential subunits have no significant changes during dry heat treatment and storage, which indicates that the vital subunits of RB protein have high stability and are not easily affected by temperature and time during dry heat treatment and storage. Zhang et al. [64] carried out RB protein analysis by electrophoresis and found that its denaturation temperature was 83.4 °C, which showed that it had good thermal stability. However, the figure’s non-reduced electrophoresis showed no significant difference in the distribution of molecular protein weight of treated RB samples during storage. Lv et al. [3] noted similar results: all protein bands had no distinct changes in RB protein after dry-heating.

The variation in results may be due to the discrepancies in protein solubility during protein extraction and the error in the amount of sample loaded by electrophoresis, which cannot be shown in the final image. Therefore, electrophoretic analysis can qualitatively evaluate the changes in RB protein during dry heat treatment and storage but cannot be precisely and quantitatively assessed. In addition, the stability of a single protein system may be more vulnerable to other factors than a complex system because of variations in processing and storage.

## 4. Conclusions

This study determined that dry heat treatment helped to enhance RB’s storage stability and shelf life. After dry heat treatment, the residual peroxidase activity and lipase activity of rice bran decreased significantly, with a maximum decrease of 90% (233 min at 120 °C); the fatty acid value and malondialdehyde content of rice bran showed an increasing trend, with a maximum increase of 31% (50 min at 130 °C). In contrast, the peroxide value decreased first and then increased. In contrast, no significant changes were observed in the fatty acid composition and crude protein content. The storage stability study of rice bran after dry heat treatment found that the fatty acid type of rice bran was unchanged, the total saturated fatty acid content and the total unsaturated fatty acid content did not change significantly, and its oleic/linoleic acid ratio increased substantially during storage with a maximum increase of 1% (88 min at 120 °C treatment). Although the amino acids of RB were lost after dry heat treatment, the content of total amino acids decreased, and the E/T and E/N to measure the nutritional value of protein were relatively stable. The electrophoretic analysis of RB protein showed that essential subunits were not affected by dry heat treatment (temperature and time) and storage, indicating that dry-heat-stabilized RB had a better storage quality.

## Figures and Tables

**Table 1:** Changes in fatty acid composition and content in dry-heat-treated rice bran.

Dry Heat Treatments (°C min)	Storage Time (d)	Myristic Acid (%)	Palmitic Acid (%)	Stearic Acid (%)	Oleic Acid (%)	Linoleic Acid (%)	Linolenic Acid (%)	Arachidic Acid (%)	Arachidonic Acid (%)	Saturated Fatty Acid (%)	Unsaturated Fatty Acid (%)	O/L
Control group	0	0.26 ± 0.02 a	16.91 ± 0.01 a	1.30 ± 0.03 a	41.61 ± 0.04 b	37.24 ± 0.12 a	0.57 ± 0.03 a	1.48 ± 0.07 a	0.63 ± 0.06 a	19.95 ± 0.07 a	80.05 ± 0.07 b	1.12 ± 0.00 a
	60	0.24 ± 0.01 a	16.08 ± 0.02 b	1.24 ± 0.00 a	42.26 ± 0.02 a	37.56 ± 0.03 a	0.55 ± 0.01 a	1.45 ± 0.02 a	0.61 ± 0.01 a	19.02± 0.05 b	80.98± 0.05 a	1.13 ± 0.00 a
120 °C 233 min	0	0.24 ± 0.00 aA	16.75 ± 0.04 aA	1.27 ± 0.03 aA	42.23 ± 0.09 aA	37.03 ± 0.06 aB	0.57 ± 0.05 aA	1.42 ± 0.01 aA	0.47 ± 0.19 aA	19.69 ± 0.01 aAB	80.31 ± 0.01 aAB	1.14 ± 0.00 aA
	60	0.24 ± 0.02 aA	16.71 ± 0.08 aA	1.30 ± 0.04 aA	42.19 ± 0.13 aA	36.94 ± 0.09 aB	0.59 ± 0.05 aA	1.42 ± 0.02 aB	0.60 ± 0.00 aB	19.67 ± 0.00 aB	80.33 ± 0.00 aB	1.14 ± 0.01 aA
120 °C 143 min	0	0.27 ± 0.01 aA	16.73 ± 0.00 aA	1.40 ± 0.13 aA	42.11 ± 0.01 aB	36.95 ± 0.02 aB	0.59 ± 0.02 aA	1.45 ± 0.05 aA	0.60 ± 0.00 aA	19.84 ± 0.12 aA	80.16 ± 0.12 aB	1.13 ± 0.00 bB
	60	0.25 ± 0.02 aA	16.74 ± 0.03 aA	1.31 ± 0.02 aA	42.19 ± 0.13 aA	36.94 ± 0.09 aB	0.59 ± 0.05 aA	1.42 ± 0.02 aAB	0.60 ± 0.00 aB	19.75 ± 0.02 aA	80.25 ± 0.02 aC	1.14 ± 0.00 aA
120 °C 88 min	0	0.27 ± 0.05 aA	16.57 ± 0.07 aB	1.26 ± 0.05 aA	41.89 ± 0.02 bB	37.35 ± 0.04 aA	0.59 ± 0.02 aA	1.46 ± 0.00 bA	0.61 ± 0.04 aA	19.56 ± 0.07 aB	80.44 ± 0.07 aA	1.12 ± 0.00 bC
	60	0.22 ± 0.01 aA	16.50 ± 0.04 aB	1.28 ± 0.03 aA	42.07 ± 0.02 aA	37.17 ± 0.03 bA	0.58 ± 0.02 aA	1.52 ± 0.01 bA	0.66 ± 0.00 abA	19.52 ± 0.01 aC	80.48 ± 0.01 aA	1.13 ± 0.00 aA
130 °C 86 min	0	0.30 ± 0.01 aA	16.61 ± 0.07 aA	1.83 ± 0.84 aA	41.68 ± 0.25 aA	36.75 ± 0.25 aA	0.58 ± 0.06 aA	1.53 ± 0.11 aA	0.71 ± 0.10 aA	20.27 ± 0.66 aA	79.73 ± 0.66 aA	1.13 ± 0.00 aA
	60	0.22 ± 0.04 aA	16.60 ± 0.02 aA	1.31 ± 0.05 aA	42.14 ± 0.03 aA	37.13 ± 0.05 aB	0.56 ± 0.02 aA	1.44 ± 0.01 aA	0.60 ± 0.01 aA	19.57 ± 0.08 aA	80.43 ± 0.08 aA	1.13 ± 0.00 aA
130 °C 66 min	0	0.26 ± 0.00 aB	16.64 ± 0.01 aA	1.27 ± 0.00 aA	41.97 ± 0.00 aA	37.24 ± 0.06 aA	0.57 ± 0.03 aA	1.45 ± 0.03 aA	0.59 ± 0.03 aA	19.62 ± 0.00 aA	80.38 ± 0.00 aA	1.13 ± 0.00 aB
	60	0.22 ± 0.01 bA	16.54 ± 0.02 bA	1.32 ± 0.11 aA	42.05 ± 0.14 aA	37.26 ± 0.03 aA	0.59 ± 0.01 aA	1.43 ± 0.01 aA	0.59 ± 0.00 aA	19.52 ± 0.15 aA	80.48 ± 0.15 aA	1.13 ± 0.00 aAB
130 °C 50 min	0	0.27 ± 0.01 aB	16.60 ± 0.04 aA	1.28 ± 0.02 aA	42.03 ± 0.03 aA	37.13 ± 0.09 aA	0.63 ± 0.05 aA	1.46 ± 0.04 aA	0.60 ± 0.03 aA	19.62 ± 0.03 aA	80.38 ± 0.03 aA	1.13 ± 0.00 aA
	60	0.24 ± 0.00 bA	16.63 ± 0.09 aA	1.31 ± 0.08 aA	41.92 ± 0.08 aA	37.28 ± 0.03 aA	0.58 ± 0.03 aA	1.44 ± 0.02 aA	0.59 ± 0.04 aA	19.63 ± 0.03 aA	80.37 ± 0.03 aA	1.12 ± 0.00 aB

**Note:** The lowercase letters in the same column refer to the significance of differences when *p* < 0.05 for different storage periods at the same temperature and the same treatment conditions; the uppercase letters in the same column refer to other treatments at the same temperature and the same storage period.

**Table 2:** Changes in crude protein content (%) in dry-heat-treated rice bran RB.

Dry Heat Treatments (°C min)	Storage Time (d)						
	0	10	20	30	40	50	60
Untreated	14.43 ± 0.04 a	14.44 ± 0.01 a	14.39 ± 0.02 a	14.38 ± 0.07 a	14.44 ± 0.14 a	14.40 ± 0.13 a	14.40 ± 0.11 a
120 °C 233 min	14.63 ± 0.13 a	14.67 ± 0.16 a	14.49 ± 0.06 a	14.66 ± 0.13 a	14.42 ± 0.08 a	14.66 ± 0.13 a	14.53 ± 0.21 a
120 °C 143 min	14.41 ± 0.10 a	14.36 ± 0.28 a	14.42 ± 0.09 a	14.42 ± 0.09 a	14.45 ± 0.23 a	14.47 ± 0.07 a	14.34 ± 0.17 a
120 °C 88 min	14.41 ± 0.10 a	14.26 ± 0.27 a	14.42 ± 0.09 a	14.38 ± 0.17 a	14.29 ± 0.17 a	14.44 ± 0.07 a	14.34 ± 0.17 a
130 °C 86 min	14.49 ± 0.03 a	14.61 ± 0.10 a	14.52 ± 0.02 a	14.36 ± 0.14 a	14.67 ± 0.21 a	14.64 ± 0.20 a	14.48 ± 0.02 a
130 °C 66 min	14.39 ± 0.15 a	14.64 ± 0.14 a	14.49 ± 0.02 a	14.45 ± 0.14 a	14.61 ± 0.21 a	14.58 ± 0.20 a	14.52 ± 0.02 a
130 °C 50 min	14.36 ± 0.15 a	14.46 ± 0.09 a	14.52 ± 0.01 a	14.46 ± 0.08 a	14.47 ± 0.04 a	14.36 ± 0.15 a	14.44 ± 0.06 a

**Note:** The lowercase letters in the same row refer to the significant difference when *p* < 0.05.

**Table 3:** Amino acid composition and content changes in dry-heat-treated rice bran at 120 and 130 °C.

	120 °C						130 °C					
Amino Acid (mg/g)	233 min		143 min		88 min		86 min		66 min		50 min	
	0 Day	60 Days	0 Day	60 Days	0 Day	60 Days	0 Day	60 Days	0 Day	60 Days	0 Day	60 Days
Aspartic acid	4.58 bB	5.09 aA	4.68 bB	5.10 aA	5.57 aA	5.04 abA	5.23 aB	4.59 cC	5.30 aB	5.25 aB	5.69 aA	5.71 aA
Threonine	1.92 aB	1.93 aA	1.95 aB	1.91 aA	2.24 aA	1.91 bA	2.20 aB	1.71 bC	2.33 aAB	1.96 cB	2.41 aA	2.16 bA
Serine	2.76 aB	2.44 cAB	2.67 aB	2.41 aB	3.01 aA	2.57 aA	2.92 bA	2.30 cC	3.04 aA	2.60 cB	3.05 aA	3.02 aA
Glutamic acid	8.15 cB	9.38 aA	8.61 aB	9.08 aA	10.27 aA	9.21 bA	9.61 aA	8.22 dC	9.12 abB	9.52 aB	9.60 aA	9.82 aA
Glycine	3.21 bA	3.45 aA	3.26 aA	3.37 aA	3.60 aA	3.47 aA	3.50 aA	3.11 bC	3.47 aA	3.50 aB	3.64 aA	3.74 aA
Alanine	3.62 cB	4.20 aA	3.57 aB	4.19 aA	3.93 aA	3.90 aA	3.91 aB	3.72 bC	4.07 aB	4.23 aB	4.33 aA	4.61 aA
Cystine	0.28 aA	0.33 aA	0.40 aA	0.28 aA	0.40 aA	0.34 aA	0.48 aA	0.26 bA	0.55 aA	0.26 bA	0.48 aA	0.26 bA
Valine	3.23 bC	3.60 aA	3.39 aB	3.49 aA	3.73 aA	3.44 aA	3.55 aB	3.07 cB	3.60 aB	3.68 aA	3.92 aA	3.60 abA
Methionine	0.24 aC	0.38 aA	0.49 aB	0.43 aA	1.03 aA	0.40 bA	0.63 abB	0.44 bC	0.81 aAB	0.48 cB	0.93 aA	0.53 dA
Isoleucine	2.27 bA	2.53 aA	2.192 cA	2.54 abA	2.43 aA	2.46 aA	2.45 aA	2.27 cA	2.46 aA	2.48 aA	2.64 aA	2.66 aA
Leucine	4.25 bB	4.47 aAB	4.07 aC	4.6 bA	4.56 aA	4.40 aB	4.60 aB	4.14 bC	4.64 aB	4.68 aB	5.01 aA	5.09 aA
Tyrosine	2.10 bB	2.29 aA	2.17 aB	2.32 aA	2.40 aA	2.19 bA	2.33 aA	2.01 bB	2.25 aA	2.28 aAB	2.45 aA	2.53 aA
Phenylalanine	2.8 bB	3.06 aA	3.02 aAB	3.10 aA	3.20 aA	2.88 bA	3.05 aB	2.71 bC	3.09 aB	3.16 aB	3.33 aA	3.32 aA
Histidine	1.786 bC	2.46 aA	2.31 bB	2.60 aA	2.59 aA	2.28 abA	1.74 bB	2.31 aA	1.93 bAB	2.69 aA	2.08 bA	2.65 aA
Lysine	3.27 bB	3.55 aA	3.28 bB	3.60 aA	3.63 aA	3.44 bA	3.42 abB	3.29 bC	3.62 aB	3.70 aB	4.10 aA	4.11 aA
Arginine	5.88 cB	6.42 abA	5.71 bC	6.43 aA	6.57 aA	6.04 abA	6.41 aA	5.46 cB	5.90 cC	6.61 aA	6.18 bB	6.77 aA
Proline	2.47 aB	2.10 aA	2.27 aB	2.30 aA	3.03 aA	2.41 bA	2.77 aB	2.05 bC	2.82 cB	2.37 aB	2.98 aA	2.55 bA
TAA	52.38 dC	57.52 bA	53.46 cB	56.99 bA	62.83 aA	57.82 bA	58.45 bB	51.69 dC	59.31 aB	59.26 aB	63.96 aA	63.53 aA
EAA	18.05 bB	19.51 aA	18.38 aB	19.69 aA	20.82 aA	18.91 abA	19.90 aB	17.63 acB	20.55 aB	20.14 aA	22.34 aA	21.46 aA
NEAA	34.33 dC	38.02 bAB	35.07 dB	37.30 cB	42.01 aA	38.90 bA	38.54 bB	34.05 dC	38.75 aB	39.12 aB	41.62 aA	42.07 aA
E/T (%)	34.46 bA	33.91 aA	34.39 aA	34.55 aA	33.14 aA	32.72 aA	34.06 aA	34.12 aA	34.66 abA	33.98 bA	34.94 aA	33.78 aA
E/N (%)	52.59 aA	51.31 abA	52.41 aA	52.80 aA	49.56 aA	48.63 aA	51.65 aA	51.79 aA	53.04 aA	51.47 aA	53.71 aA	51.02 aA

**Note:** The lowercase letters in the same row refer to the significant difference when *p* < 0.05 for different storage periods at the same temperature and the same treatment conditions; the uppercase letters in the same row refer to the *p* values of varying treatment times at the same temperature and the same storage period.

## Data Availability

The data presented in this study are available on request from the corresponding author.

## Supplementary Materials

The following supporting information can be downloaded at: https://www.mdpi.com/article/10.3390/foods11244001/s1, Table S1: Changes in amino acid composition and content in the control group; Table S2: Changes of amino acid composition and content in dry heat-treated rice bran at 120 °C; Table S3 Changes of amino acid composition and content in dry heat-treated rice bran at 130 °C.

## Abbreviations

AAC	Amino acid composition
AAs	Amino acids
AOAC	Association of Official Agricultural Chemists
CNT	Control
FAO	The Food and Agriculture Organization
FAC	Fatty acid composition
FFA	Free fatty acids
PRA	Peroxidase residual activity
HCl	Hydrochloric Acid
IR	Infrared
LA	lipase activity
MDA	malondialdehyde content
PV	Peroxide value
RB	Rice bran
SDS-PAGE	Sodium dodecyl sulfate-polyacrylamide gel electrophoresis
UFA	Unsaturated fatty acid
WHO	World Health Organization
