# Supplementary material for: The Effects of Thermal Treatment on Lipid Oxidation, Protein Changes, and Storage Stabilization of Rice Bran
Source: Foods. 2022 Dec 10;11(24):4001. doi: 10.3390/foods11244001 (PMC9778295; doi:10.3390/foods11244001)
Supplement: Supplementary file 1 [file foods-11-04001-s001.zip › foods-2088489-supplementary.pdf]

Supplementary data

Table S1. Changes in amino acid composition and content in the control group.

Amino acid (mg/g)	Control group			
	**0days**	**10 days**	**30 days**	**60 days**
Aspartic acid	5.00 ± 0.11a	4.94 ± 0.25a	5.02 ± 0.15a	5.03 ± 0.05a
Threonine	1.96 ± 0.09a	1.99 ± 0.02a	1.98 ± 0.03a	1.92 ± 0.05a
Serine	2.80 ± 0.05a	2.79 ± 0.03a	2.51 ± 0.02b	2.53 ± 0.04b
Glutamic acid	9.10 ± 0.17a	9.05 ± 0.39a	9.24 ± 0.16a	9.17 ± 0.02a
Glycine	3.29 ± 0.02a	3.30 ± 0.07a	3.36 ± 0.07a	3.31 ± 0.01a
Alanine	3.80 ± 0.11a	3.37 ± 0.17a	3.90 ± 0.08a	3.88 ± 0.03a
Cystine	0.40 ± 0.01a	0.32 ± 0.21a	0.46 ± 0.03a	0.44 ± 0.02a
Valine	3.37 ± 0.04b	3.34 ± 0.07b	3.53 ± 0.03a	3.56 ± 0.02a
Methionine	0.56 ± 0.21a	0.40 ± 0.35a	0.53 ± 0.07a	0.44 ± 0.13a
Isoleucine	2.42 ± 0.02a	2.39 ± 0.17a	2.53 ± 0.03a	2.49 ± 0.02a
Leucine	4.22 ± 0.04b	4.23 ± 0.04ab	4.34 ± 0.02a	4.33 ± 0.02ab
Tyrosine	2.25 ± 0.03a	2.18 ± 0.09a	2.26 ± 0.03a	2.22 ± 0.02a
Phenylalanine	2.98 ± 0.05a	2.96 ± 0.09a	3.03 ± 0.02a	3.01 ± 0.05a
Histidine	1.85 ± 0.11a	2.33 ± 0.16a	2.36 ± 0.09a	2.30 ± 0.32a
Lysine	3.47 ± 0.06a	3.44 ± 0.16a	3.51 ± 0.01a	3.60 ± 0.01a
Arginine	6.31 ± 0.03a	6.27 ± 0.36a	6.44 ± 0.03a	6.45 ± 0.03a
Proline	2.64 ±0.04a	2.67 ± 0.09a	2.39 ± 0.01b	2.25 ± 0.04b
TAA	56.63 ± 0.03b	55.20 ± 0.64c	58.58 ± 0.16a	55.90 ± 0.11bc
EAA	18.97 ± 0.36a	18.75 ± 0.91a	19.45 ± 0.23a	19.35 ± 0.61a
NEAA	37.66 ± 0.45b	36.45 ± 0.26c	39.13 ± 0.38a	36.55 ± 0.50bc
E/T(%)	33.50 ± 0.71a	33.96 ± 1.25a	33.21 ± 0.48a	34.62 ± 1.02a
E/N(%)	50.39 ± 1.60a	51.44 ± 2.86a	49.72 ± 1.07a	52.96 ± 2.38a

Note: The lowercase letters in the same row refer to the significant difference when $p<0.05$.

Table S2. Changes of amino acid composition and content in dry heat-treated rice bran at 120°C.

**Amino acid (mg/g)**	**120°C 233 min**				**120°C 143 min**				**120°C for 88 min**			
	**0day**	**10 days**	**30 days**	**60 days**	**0day**	**10 days**	**30 days**	**60 days**	**0day**	**10 days**	**30 days**	**60 days**
Aspartic acid	4.58 ± 0.04bB	4.65 ± 0.03bA	5.10 ± 0.00aA	5.09 ± 0.00aA	4.68 ± 0.08bB	4.82 ± 0.04abA	5.06 ± 0.11aA	5.10 ± 0.06aA	5.57 ± 0.07aA	4.85 ± 0.28bA	4.95 ± 0.10bA	5.04 ± 0.09abA
Threonine	1.92 ± 0.01aB	1.97 ± 0.03aA	1.93 ± 0.00aA	1.93 ± 0.06aA	1.95 ± 0.05aB	1.99 ± 0.18aA	1.95 ± 0.02aA	1.91 ± 0.05aA	2.24 ± 0.04aA	1.88 ± 0.13bA	1.85 ± 0.04bA	1.91 ± 0.02bA
Serine	2.76 ± 0.05aB	2.78 ± 0.02aA	2.57 ± 0.01bA	2.44 ± 0.02cAB	2.67 ± 0.03aB	2.79 ± 0.43aA	2.61 ± 0.06aA	2.41 ± 0.02aB	3.01 ± 0.04aA	2.62 ± 0.14aA	2.58 ± 0.18aA	2.57 ± 0.03aA
Glutamic acid	8.15 ± 0.04cB	8.66 ± 0.04bA	9.39 ± 0.06aA	9.38 ± 0.05aA	8.61 ± 0.37aB	9.02 ± 0.05aA	9.29 ± 0.32aA	9.08 ± 0.08aA	10.27 ± 0.07aA	8.66 ± 0.38Ab	8.91 ± 0.07bA	9.21 ± 0.13bA
Glycine	3.21 ± 0.04bA	3.23 ± 0.03bA	3.48 ± 0.02aA	3.45 ± 0.02aA	3.26 ± 0.05aA	3.35 ± 0.05aA	3.39 ± 0.12aA	3.37 ± 0.02aA	3.60 ± 0.25aA	3.44 ± 0.40aA	3.38 ± 0.11aA	3.47 ± 0.02aA
Alanine	3.62 ± 0.04cB	3.61 ± 0.06cA	4.09 ± 0.02bAB	4.20 ± 0.01aA	3.57 ± 0.03aB	3.92 ± 0.02aA	4.18 ± 0.05aA	4.19 ± 0.03aA	3.93 ± 0.14aA	3.86 ± 0.39Aa	3.84 ± 0.07aB	3.90 ± 0.16aA
Cystine	0.28 ± 0.08aA	0.23 ± 0.11aA	0.34 ± 0.01aA	0.33 ± 0.05aA	0.40 ± 0.06aA	0.38 ± 0.08aA	0.34 ± 0.04aA	0.28 ± 0.08aA	0.40 ± 0.03aA	0.35 ± 0.13aA	0.34 ± 0.01aA	0.34 ± 0.02aA
Valine	3.23 ± 0.05bC	3.28 ± 0.06bA	3.59 ± 0.04aA	3.60 ± 0.02aA	3.39 ± 0.03aB	3.41 ± 0.06aA	3.57 ± 0.16aA	3.49 ± 0.14aA	3.73 ± 0.06aA	3.41 ± 0.28aA	3.44 ± 0.11aA	3.44 ± 0.05aA
Methionine	0.24 ± 0.07aC	0.23 ± 0.05aA	0.33 ± 0.02aA	0.38 ± 0.02aA	0.49 ± 0.06aB	0.50 ± 0.12aA	0.42 ± 0.01aA	0.43 ± 0.02aA	1.03 ± 0.07aA	0.38 ± 0.14bA	0.33 ± 0.06bA	0.40 ± 0.01bA
Isoleucine	2.27 ± 0.07bA	2.29 ± 0.07bA	2.51 ± 0.02aA	2.53 ± 0.00aA	2.19 ± 0.02cA	2.32 ± 0.02bcA	2.59 ± 0.06aA	2.54 ± 0.06abA	2.43 ± 0.15aA	2.34 ± 0.18aA	2.38 ± 0.02aA	2.46 ± 0.02aA
Leucine	4.25 ± 0.05bB	4.23 ± 0.01bA	4.47 ± 0.02aA	4.47 ± 0.02aAB	4.07 ± 0.07aC	4.54 ± 0.11bA	4.66 ± 0.11bA	4.6 ± 0.06bA	4.56 ± 0.03aA	4.30 ± 0.31aA	4.37 ± 0.14aA	4.40 ± 0.01aB
Tyrosine	2.10 ± 0.01bB	2.11 ± 0.01bA	2.28 ± 0.01aA	2.29 ± 0.00aA	2.17 ± 0.08aB	2.25 ± 0.08aA	2.30 ± 0.07aA	2.32 ± 0.04aA	2.40 ± 0.02aA	2.17 ± 0.10bA	2.14 ± 0.01bA	2.19 ± 0.01bA
Phenylalanine	2.88 ± 0.01bB	2.94 ± 0.01bA	3.06 ± 0.03aA	3.06 ± 0.03aA	3.02 ± 0.15aAB	3.03 ± 0.13aA	3.15 ± 0.10aA	3.10 ± 0.06aA	3.20 ± 0.05aA	2.83 ± 0.04bA	2.84 ± 0.03bA	2.88 ± 0.02bA
Histidine	1.78 ± 0.06bC	2.33 ± 0.01aA	2.50 ± 0.06aAB	2.46 ± 0.09aA	2.31 ± 0.01bB	2.52 ± 0.05abA	2.66 ± 0.05aA	2.60 ± 0.06aA	2.59 ± 0.05aA	2.22 ± 0.23bA	2.26 ± 0.01abB	2.28 ± 0.05abA
Lysine	3.27 ± 0.06bB	3.35 ± 0.01bA	3.54 ± 0.01aA	3.55 ± 0.03aA	3.28 ± 0.04bB	3.32 ± 0.05bA	3.60 ± 0.01aA	3.60 ± 0.02aA	3.63 ± 0.02aA	3.36 ± 0.10bA	3.41 ± 0.01bB	3.44 ± 0.03bA
Arginine	5.88 ± 0.04cB	5.98 ± 0.01bcA	6.46 ± 0.11aA	6.42 ± 0.12abA	5.71 ± 0.04bC	6.39 ± 0.10aA	6.47 ± 0.16aA	6.43 ± 0.13aA	6.57 ± 0.05aA	5.96 ± 0.36bA	6.06 ± 0.05abA	6.04 ± 0.10abA
Proline	2.47 ± 0.21aB	2.56 ± 0.19aA	2.66 ± 0.12aA	2.10 ± 0.08aA	2.27 ± 0.17aB	2.49 ± 0.20aA	2.45 ± 0.03aA	2.30 ± 0.14aA	3.03 ± 0.06aA	2.47 ± 0.13bA	2.29 ± 0.10bA	2.41 ± 0.07bA
TAA	52.38 ± 0.13dC	54.80 ± 0.31cB	59.14 ± 0.20aB	57.52 ± 0.52bA	53.46 ± 0.30cB	57.69 ± 0.23bA	60.61 ± 0.17aA	56.99 ± 0.99bA	62.83 ± 0.30aA	53.34 ± 0.44cC	54.08 ± 0.34cC	57.82 ± 0.41bA
EAA	18.05 ± 0.19bB	18.29 ± 0.10bA	19.43 ± 0.23aA	19.51 ± 0.13aA	18.38 ± 0.32aB	19.11 ± 0.33aA	19.94 ± 0.89aA	19.69 ± 0.81aA	20.82 ± 0.43aA	18.50 ± 0.93bA	18.62 ± 0.77abA	18.91 ± 0.33abA

NEAA	34.33 ± 0.32dC	36.51 ± 0.41cB	39.70 ± 0.43aA	38.02 ± 0.39bAB	35.07 ± 0.02dB	38.58 ± 0.10bA	40.68 ± 0.71aA	37.30 ± 0.17cB	42.01 ± 0.13aA	34.84 ± 0.49cC	35.46 ± 0.43cB	38.90 ± 0.74bA
E/T(%)	34.46 ± 0.46bA	33.38 ± 0.37aA	32.86 ± 0.50aA	33.91 ± 0.07aA	34.39 ± 0.41aA	33.13 ± 0.44aA	32.89 ± 1.37aA	34.55 ± 0.83aA	33.14 ± 0.52aA	34.68 ± 1.46aA	34.43 ± 1.21aA	32.72 ± 0.80aA
E/N(%)	52.59 ± 1.06aA	50.11 ± 0.83bA	48.95 ± 1.11bA	51.31 ± 0.17abA	52.41 ± 0.94aA	49.54 ± .98aA	49.04 ± 3.04aA	52.80 ± 1.94aA	49.56 ± 1.16aA	53.14 ± 3.42aA	52.54 ± 2.82aA	48.63 ± 1.77aA

Table S3. Changes of amino acid composition and content in dry heat-treated rice bran at 130°C.

Amino acid (mg/g)	130°C 86 min				130°C 66 min				130°C for 50 min			
	**0day**	**10 days**	**30 days**	**60 days**	**0day**	**10 days**	**30 days**	**60 days**	**0day**	**10 days**	**30 days**	**60 days**
Aspartic acid	5.23 ± 0.07aB	5.18 ± 0.07aA	4.80 ± 0.04bC	4.59 ± 0.01cC	5.30 ± 0.02aB	5.24 ± 0.15aA	5.27 ± 0.02aB	5.25 ± 0.01aB	5.69 ± 0.06aA	5.46 ± 0.48aA	5.66 ± 0.02aA	5.71 ± 0.04aA
Threonine	2.20 ± 0.10aB	2.22 ± 0.05aA	1.75 ± 0.01bC	1.71 ± 0.02bC	2.33 ± 0.02aAB	2.12 ± 0.01bA	1.98 ± 0.01cB	1.96 ± 0.01cB	2.41 ± 0.01aA	2.14 ± 0.11bA	2.17 ± 0.02bA	2.16 ± 0.03bA
Serine	2.92 ± 0.06bA	3.10 ± 0.09aA	2.32 ± 0.02cB	2.30 ± 0.01cC	3.04 ± 0.02aA	2.92 ± 0.03bA	2.60 ± 0.02cAB	2.60 ± 0.01cB	3.05 ± 0.08aA	2.99 ± 0.17aA	2.92 ± 0.13aA	3.02 ± 0.05aA
Glutamic acid	9.61 ± 0.04aA	9.38 ± 0.03bA	8.34 ± 0.02cC	8.22 ± 0.01dC	9.12 ± 0.06abB	8.89 ± 0.35bA	9.35 ± 0.02abB	9.52 ± 0.01aB	9.60 ± 0.02aA	9.45 ± 0.60aA	9.61 ± 0.02aA	9.82 ± 0.01aA
Glycine	3.50 ± 0.04aA	3.51 ± 0.02aA	3.12 ± 0.04bB	3.11 ± 0.01bC	3.47 ± 0.05aA	3.41 ± 0.10aA	3.46 ± 0.01aA	3.50 ± 0.01aB	3.64 ± 0.17aA	3.55 ± 0.24aA	3.68 ± 0.04aA	3.74 ± 0.05aA
Alanine	3.91 ± 0.01aB	3.93 ± 0.01aA	3.75 ± 0.01bB	3.72 ± 0.02bC	4.07 ± 0.07aB	4.10 ± 0.15aA	4.21 ± 0.03aA	4.23 ± 0.02aB	4.33 ± 0.09aA	4.15 ± 0.43aA	4.37 ± 0.03aA	4.61 ± 0.01aA
Cystine	0.48 ± 0.02aA	0.45 ± 0.06aA	0.52 ± 0.03aA	0.26 ± 0.02bA	0.55 ± 0.02aA	0.53 ± 0.08aA	0.55 ± 0.03aA	0.26 ± 0.01bA	0.48 ± 0.04aA	0.49 ± 0.04aA	0.26 ± 0.00bB	0.26 ± 0.00bA
Valine	3.55 ± 0.03aB	3.51 ± 0.01aA	3.29 ± 0.01bB	3.07 ± 0.03cB	3.60 ± 0.16aB	3.58 ± 0.16aA	3.67 ± 0.02aA	3.68 ± 0.01aA	3.92 ± 0.01aA	3.51 ± 0.23bA	3.58 ± 0.07abA	3.60 ± 0.04abA
Methionine	0.63 ± 0.12abB	0.73 ± 0.07aA	0.59 ± 0.00abB	0.44 ± 0.01bC	0.81 ± 0.02aAB	0.83 ± 0.01aA	0.70 ± 0.01bB	0.48 ± 0.00cB	0.93 ± 0.02aA	0.80 ± 0.02bA	0.71 ± 0.00cB	0.53 ± 0.00dA
Isoleucine	2.45 ± 0.03aA	2.41 ± 0.02abA	2.38 ± 0.01bA	2.27 ± 0.00cA	2.46 ± 0.07aA	2.43 ± 0.14aA	2.41 ± 0.00aA	2.48 ± 0.06aA	2.64 ± 0.07aA	2.58 ± 0.35aA	2.62 ± 0.09aA	2.66 ± 0.13aA
Leucine	4.60 ± 0.05aB	4.65 ± 0.01aA	4.15 ± 0.00bB	4.14 ± 0.00bC	4.64 ± 0.06aB	4.58± 0.21aA	4.64 ± 0.11aB	4.68 ± 0.05aB	5.01 ± 0.02aA	4.80 ± 0.51aA	4.96 ± 0.01aB	5.09 ± 0.05aA
Tyrosine	2.33 ± 0.01aA	2.32 ± 0.00aA	2.23 ± 0.07aA	2.01 ± 0.07bB	2.25 ± 0.01aA	2.27 ± 0.18aA	2.25 ± 0.05aA	2.28 ± 0.06aAB	2.45 ± 0.15aA	2.41 ± 0.24aA	2.49 ± 0.01aA	2.53 ± 0.06aA
Phenylalanine	3.05 ± 0.03aB	3.05 ± 0.06aA	2.79 ± 0.00bC	2.71 ± 0.00bC	3.09 ± 0.03aB	3.08 ± 0.20aA	3.07 ± 0.03aB	3.16 ± 0.03aB	3.33 ± 0.05aA	3.21 ± 0.26aA	3.31 ± 0.00aA	3.32 ± 0.00aA
Histidine	1.74 ± 0.00bB	2.39 ± 0.12aA	2.34 ± 0.02aB	2.31 ± 0.13aA	1.93 ± 0.05bAB	1.88 ± 0.09bB	2.62 ± 0.08aAB	2.69 ± 0.06aA	2.08 ± 0.13bA	1.99 ± 0.17bAB	2.67 ± 0.00aA	2.65 ± 0.02aA
Lysine	3.42 ± 0.04abB	3.51 ± 0.13aA	3.32 ± 0.00abC	3.29 ± 0.00bC	3.62 ± 0.09aB	3.61 ± 0.08aA	3.67 ± 0.02aB	3.70 ± 0.07aB	4.10 ± 0.12aA	3.84 ± 0.53aA	4.04 ± 0.02aA	4.11 ± 0.07aA
Arginine	6.41 ± 0.02aA	6.41 ± 0.02aA	5.66 ± 0.04bC	5.46 ± 0.02cB	5.90 ± 0.02cC	5.85 ± 0.01cC	6.17 ± 0.02bB	6.61 ± 0.01aA	6.18 ± 0.02bB	6.20 ± 0.02bB	6.83 ± 0.11aA	6.77 ± 0.09aA
Proline	2.77 ± 0.03aB	2.77 ± 0.03aA	2.03 ± 0.01bC	2.05 ± 0.00bC	2.82 ± 0.03cB	2.49 ± 0.04cB	2.33 ± 0.01bB	2.37 ± 0.02aB	2.98 ± 0.01aA	2.88 ± 0.16aA	2.56 ± 0.04bA	2.55 ± 0.03bA
TAA	58.45 ± 0.19bB	59.82 ± 0.34aA	53.35 ± 0.29cC	51.69 ± 0.42dC	59.31 ± 0.06aB	56.82 ± 0.53bB	58.96 ± 0.24aB	59.26 ± 0.97aB	63.96 ± 0.78aA	57.66 ± 0.49bB	62.78 ± 0.49aA	63.53 ± 0.19aA
EAA	19.90 ± 0.21aB	20.08 ± 0.33aA	18.27 ± 0.04abC	17.63 ± 0.00acB	20.55 ± 0.23aB	20.24 ± 0.77aA	20.14 ± 0.16aC	20.14 ± 0.38aA	22.34 ± 0.30aA	20.90 ± 2.00aA	21.39 ± 0.30aA	21.46 ± 0.65aA

NEAA	38.54 ± 0.41bB	39.75 ± 0.01aA	35.08 ± 0.25cC	34.05 ± 0.41dC	38.75 ± 0.29aB	36.59 ± 0.24bB	38.82 ± 0.08aB	39.12 ± 0.60aB	41.62 ± 1.08aA	36.76 ± 1.52bB	41.39 ± 0.78aA	42.07 ± 0.46aA
E/T(%)	34.06 ± 0.48aA	33.56 ± 0.36aA	34.24 ± 0.11aA	34.12 ± 0.27aA	34.66 ± 0.42abA	35.61 ± 1.03aA	34.16 ± 0.14abA	33.98 ± 0.08bA	34.94 ± 0.90aA	36.24 ± 3.17aA	34.07 ± 0.74aA	33.78 ± 0.92aA
E/N(%)	51.65 ± 1.10aA	50.51 ± 0.83aA	52.07 ± 0.26aA	51.79 ± 0.62aA	53.04 ± 0.99aA	55.31 ± 2.48aA	51.89 ± 0.32aA	51.47 ± 0.18aA	53.71 ± 2.12aA	57.02 ± 7.81aA	51.68 ± 1.69aA	51.02 ± 2.10aA
